# Effect of Implant Positions and Angulations on Retentive Strength of 2-Implant Mandibular Overdentures: An In Vitro Study with the New 3D-Printed Simulation Method

**DOI:** 10.1155/2022/7052955

**Published:** 2022-09-14

**Authors:** Pravinkumar G. Patil, Liang Lin Seow, Rashmi Uddanwadikar, Allan Pau, Piyush D. Ukey

**Affiliations:** ^1^Division of Restorative Dentistry, School of Dentistry, International Medical University, Kuala Lumpur, Malaysia; ^2^Department of Mechanical Engineering, Visvesvaraya National Institute of Technology, Nagpur, India; ^3^Division of Clinical Oral Health Sciences, School of Dentistry, International Medical University, Kuala Lumpur, Malaysia; ^4^NU OSSA Mediquip Pvt Ltd, Nagpur, India

## Abstract

**Objectives:**

To evaluate the retentive strength of overdenture attachments in 2-implant mandibular overdenture (2IMO) with implants placed at different positions and angulations.

**Materials and Methods:**

Edentulous mandibular models were 3D-printed using CBCT images and Materialise Mimics software and the denture models using the intraoral scanner. Two standard implants were placed parallel at different positions from midline (5, 10, 15, and 20 mm) with 0-0 degree angulations and with different distal angulations (0–5, 0–10, 0–15, 5-5, 10-10, and 15-15 degrees) at 10±mm from midline representing 10 study groups. Low-profile male attachments were attached to the implants and the female pink attachments were picked up in the denture. A total of 4 simulated overdenture model sets for each of the 10 study groups were subjected to the universal testing machine thrice to measure a peak load (N) to disengage the attachments vertically. Data were analyzed using one-way ANOVA and Tukey's post hoc test at 0.05 significance level.

**Results:**

Varying implant positions had a statistically significant effect on the retentive strengths of the attachments (*F* = 5.61, *P* = 0.002). Peak load-to-dislodgement values (in increasing order) were 49.64 ± 8.27 N for 5 mm, 53.26 ± 11.48 N for 10 mm, 60.24 ± 12.31 N for 15 mm, and 64.80 ± 6.78 N for 20 mm groups. The retentive strength of the 20 mm group was significantly higher than 5 mm (*P* = 0.003) and 10 mm (*P* = 0.03) groups. Varying implant angulations had a significant effect on the retentive strengths of the attachments (*F* = 7.412, *P* = 0.000). The peak load-to-dislodgement values (in increasing order) were 48.20 ± 15.59 N for 5-5 degrees, 53.26 ± 11.48 N for 0-0 degrees, 54.96 ± 8.25 N for 0–5 degrees, 57.71 ± 7.62 N for 10-10 degrees, 66.00 ± 17.54 N for 15-15 degrees, 66.18 ± 14.09 N for 0–10 degrees, and 77.38 ± 10.33 N for 0–15 degrees. Retentive strength of 0–15 degrees was significantly (*P* < 0.05) higher than those of 0-0, 0–5, 5-5, and 10-10 degrees and that of 5-5 degrees was significantly (*P* < 0.05) lower than those of 0–10, 0–15, and 15-15 groups.

**Conclusions:**

Retentive strength of the 2IMO increased with increase in distance of implants from midline and increased with increase in distal angulations.

## 1. Introduction

In complete denture users, a stable mandibular denture is the most critical factor for their satisfaction [1]. The 2-implant mandibular overdenture (2IMO) is a popular treatment for the edentulous mandible that can enhance the retention and stability of the denture at a greater extent [2]. The implant overdenture is an assembly of different components including the denture, mucosa, bone, implant, and attachments. The overdenture attachment is one of the most influential factors in maintaining the long-term success of the implant overdentures. The stud attachments have been popular in practice (as opposed to the bar attachments) and include conventional ball attachments and newer low-profile self-aligning attachments (LOCATOR; Zest Anchors, Equator; Rhein83, and ERA; Sterngold) [3, 4]. The flexible matrix components of these attachments are manufactured with the range of retentive strengths and are differentiated with different colors. The authors are unaware of specific guidelines to select the specific retentive strength of these attachments in different clinical situations. Moreover, in 2IMO, there is always a possibility of 2 implants being placed at different positions in the arch from midline and at different angulations. The researchers have found out the differences in the retentive strengths of these attachments by changing implant positions [5], heights [6], and angulations [7–10].

Scherer et al. [5] evaluated the effect of implant positions at lateral incisor, canine, 1st, and 2nd premolar on the in vitro retention and stability of a simulated 2IMO and concluded that the retention and stability are increased with the distal implant positions up to the second premolar. Sia et al. [6] evaluated the effect of the differential heights of pairs of the LOCATOR attachments (0, 2, 4, and 6 mm) on the retention of 2IMO after 6 months of simulated function and concluded that varying the heights of the LOCATOR attachments had a statistically significant effect on the retentive strengths of the pink LOCATOR attachments. Al-Ghafli et al. [7] investigated the effect of cyclic dislodgement on the retention of an overdenture attachment system when 2 implants were placed at angulations of 0, 5, 10, 15, and 20 degrees and concluded that implant angulations negatively affect attachment retention longevity. Elsyad et al. [8] evaluated the influence of labial implant inclination on the retention and stability of different resilient stud attachments for 2IMO. Two implants were inserted at the canine areas with 0, 10, 20, and 30 degrees of labial inclination in 4 identical overdenture models using regular retentive inserts and it was found out that the inclination of 30° recorded the highest retention, and the inclination of 20° showed the lowest retention. Elsyad et al. [9] also evaluated the effect of distal implant inclination on dislodging forces of different LOCATOR attachments on 4 similar models with 2 implants placed in the canine region at 0, 5, 10, and 20 degrees of distal inclinations. Axial and nonaxial (anterior, posterior, and lateral) retentive forces were measured initially and after 540 cycles of denture insertion and removal. The retention of LOCATOR attachments was significantly affected by the degree of distal implant inclination and the type of nylon inserts. The LOCATOR medium retention is recommended to retain overdentures when implants have 5 or 10 degrees of distal inclination, and extra light and light retentions were recommended with 20-degree inclination to maintain high axial and nonaxial retention after wear. Kobayashi et al. [10] compared the change in the retentive force and removal torque of three attachment systems in 2IMO (with implants placed at 0 and 12 degrees of angulations) under simulated conditions of total 14,600 insertion-removal cycles in 0.9% sodium chloride solution. They concluded that the Dalbo®-Plus and SFI® Bar exhibit higher retentive capacities than the LOCATOR® attachment over time. An angulation of up to 12 degrees between implants does not seem to have a significant effect on retentive forces and attachment wear. Most of these studies have used different numbers of insertion-removal cycles to simulate long-term oral function to indicate the attachment longevity in addition to their retentive strengths.

Many investors evaluated the effect of cyclic loading on stud attachment wear [9, 11–15] and initial retention was significantly higher than retention after wear or simulated clinical function under different number of insertion-removal cycles for different type of inserts. However, the literature lacks the information on the retentive strength of the attachments without insertion-removal cycles which simulates the overdenture usage from the first day. Patil et al. [16] and Patil et al. [17] have carried out systematic reviews on the effect of different unsplinted attachments on peri-implant outcomes [16] and the patient-reported outcome measures (PROMs) [17] in 2IMOs and concluded that the unsplinted (ball and low-profile) attachments have no influence on PROMs in the normal interarch space. The gingival and bleeding index of the patients were not influenced by any of the unsplinted attachments (stud, magnet, and telescopic) studied [16]. Most of these studies [11–17] used canine position as a standard position in 2IMO patients. The literature lacks information regarding their clinical performance especially when the implants were placed at different positions or angulations.

The purpose of this laboratory study, using 3D-printed-simulation models, was to evaluate the retentive strengths of the 2IMO with different implant positions and angulations so that the appropriate attachment strength can be selected to optimize the overall retention. The research hypotheses were that the implant positions, as indicated by increase in the implant distance from midline, result in higher retentive strength of the LOCATOR attachments retaining 2IMO and increase in the interimplant angulation results in higher retentive strength of the LOCATOR attachments retaining 2IMO.

## 2. Materials and Methods

**Figure 1:**
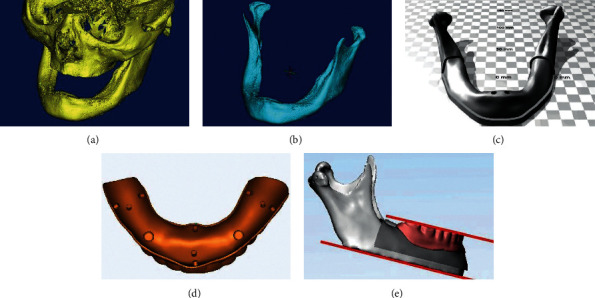
3D modeling of mandible and denture. (a) DICOM file of the skull and mandible. (b) Mandible extracted from the skull. (c) Representative 3D mandibular model of the 5 mm group. (d) Representative 3D denture model of the 20 mm group. (e) Complete overdenture model with base flattened parallel to denture occlusal plane.

Institutional ethical committee approval was obtained for this study (Project ID: R 216-2018). A written informed consent was obtained from a 59-year-old completely edentulous man (with 5 years of denture wearing experience) and he was scanned by using a cone beam computed tomography (CBCT). The digital imaging and communications in medicine (DICOM) files were imported into a 3D image design and processing software program (Mimics; Materialise) (Figure 1(a)). The mandible was extracted (Figure 1(b)) and imported to a 3D modeling software program (3-matic; Materialise) to refine the surfaces. Surface offset of 2 mm was provided to the mandibular alveolar region (Figure 1(c)) to accommodate soft mucosal layer. The implant osteotomy holes were created virtually in 3-matic software program (to facilitate placement of 4.3 × 13 mm implants) (Figure 1(c)) in 3D mandibular models at different positions from midline (5, 10, 15, and 20 mm) at 0-0 degree angulation and with different distal angulations (0–5, 0–10, 0–15, 5-5, 10-10, and 15-15 degrees) at 10 mm distance from midline representing 10 study groups. A mandibular denture of the same patient was scanned extraorally from all surfaces with an intraoral scanner (TRIOS; 3Shape A/S). The DICOM files of the denture were imported to the 3-matic software program, and the tissue surface was adjusted and 9 stops were created so that the denture was away from the mandibular surface by 2 mm to simulate uniform mucosal layer (Figure 1(d)). The base of the mandible was flattened and made parallel with denture occlusal plane (Figure 1(e)). The standard tessellation language (STL) files of the mandible and the denture were imported for 3D printing to obtain the physical models.

**Figure 2:**
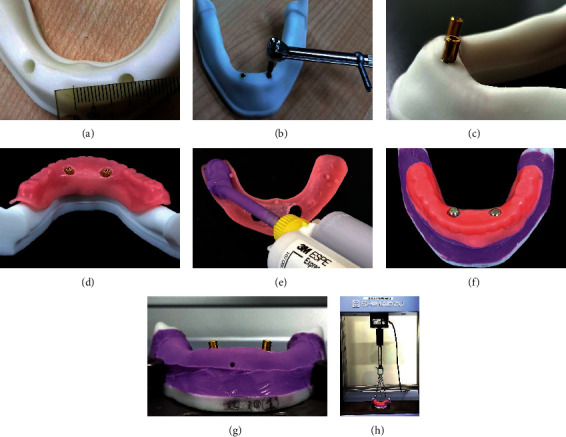
Methodology of 2IMO simulation using 3D-printed models. (a) Representative 3D mandibular model of the 15 mm group. (b) Implant placement in predetermined holes. (c) LOCATOR male attachment placed onto implants. (d) Holes created in denture corresponding to area of attachments. (e) Soft-tissue layer simulated using VPS material. (f) Denture ready to pick up female attachment using autopolymerizing acrylic resin. (g) The mandibular 3D model with 10-10 degree angulation attached to a universal testing machine. (h) Vertical pull force applied to dislodge.

The mandibles were printed using white colored 3D-printed resin (3D printing UV Sensitive Resin; ANYCUBIC) in a printer (PHOTON MONO X; ANYCUBIC) according to the manufacturer's instructions (Figure 2(a)). The dentures were printed using pink-colored 3D-printed resin (NextDent Denture 3D+; NextDent) in a printer (NextDent 5100 3D Printer; NextDent). A total of 40 sets of the 3D-printed mandibles (4 per study group) and 40 dentures were obtained. Two standard dummy implants (Nobel Active; Nobel Biocare) (4.3 × 13 mm) were placed in each model in the predetermined virtually planned holes (Figure 2(b)). The LOCATOR attachment (Zest Anchors) (4 mm height) was placed onto the implants (Figure 2(c)). Uniform 2 mm thick mucosal layer was simulated using a light body vinyl polysiloxane (VPS) impression material (Express XT Light body; 3M ESPE) with the help of tissue stops of the 3D-printed denture (Figures 2(d) and 2(e)). Two male processing units were placed onto the LOCATOR male attachments (Figure 2(f)) and the female attachments (Pink) were picked up in the denture using autopolymerizing acrylic resin.

Complete sets of simulated 2IMOs were developed for 10 specific conditions representing 10 different groups as described above. Individual set of 2IMO was attached to the universal testing machine (Shimadzu Corporation) (Figure 2(g)). A total of 3 self-tapping hooks were fixed in the denture (2 at 2nd molar and 1 at mid-incisor area) and each hook was connected with a metal chain and all 3 chains further connected with a single metal ring attached to the upper moving arm of the testing machine (Figure 2(h)). All 3 chains were manually evaluated for uniform tightness and adjusted using self-tapping hooks at the beginning of the pull test to ensure uniform pulling force from all 3 areas. The testing machine was calibrated and balanced by using the testing machine's computer algorithm. A vertical pull was used to determine the retention against a vertically directed dislodging force parallel to the path of insertion at a constant crosshead speed of 50.8 mm/min [5]. The force was applied until the prosthesis was separated from both attachments and a peak load (N) was recorded (Figure 2(h)).

Four identical simulated 2IMO sets (representing each study group) were subjected to perform 3 pull tests each, to record total 12 retentive strength values per group under universal testing machine. The data were analyzed using one-way ANOVA and Tukey's post hoc test at 0.05 significance level.

## 3. Results

**Figure 3:**
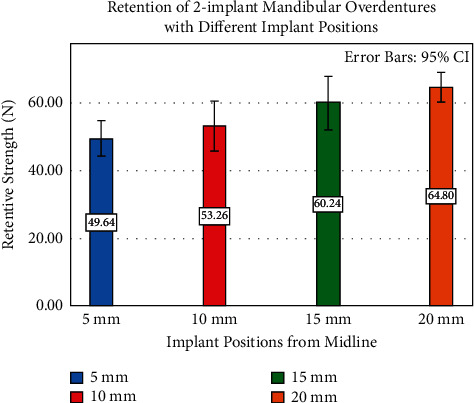
Retention of 2IMO with different implant positions.

**Figure 4:**
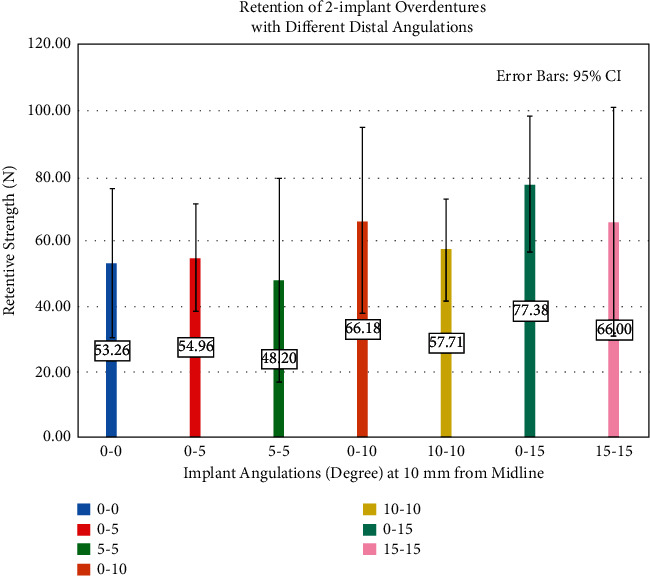
Retention of 2IMO with different implant angulations.

Descriptive statistics of retention strength results with different positions of implants are described in Table 1. Varying implant positions had a statistically significant effect on the retentive strengths of the attachments (*F* = 5.61,*P*=0.002) (Table 2). Peak load-to-dislodgement values (in increasing order) were 49.64 ± 8.27 N for 5 mm, 53.26 ± 11.48 N for 10 mm, 60.24 ± 12.31 N for 15 mm, and 64.80 ± 6.78 N for 20 mm group (Figure 3). The retentive strength of 20 mm group was significantly higher than those of 5 mm (*P*=0.003) and 10 mm (*P*=0.03) groups (Table 3). Descriptive statistics of retention strength results with different angulations of the implants are described in Table 4. Varying implant angulations had a significant effect on the retentive strengths of the attachments (*F* = 7.412, *P*=0.000) (Table 5). The peak load-to-dislodgement values (in increasing order) were 48.20 ± 15.59 N for 5-5 degrees, 53.26 ± 11.48 N for 0-0 degrees, 54.96 ± 8.25 N for 0–5 degrees, 57.71 ± 7.62 N for 10-10 degrees, 66.00 ± 17.54 N for 15-15 degrees, 66.18 ± 14.09 N for 0–10 degrees, and 77.38 ± 10.33 N for 0–15 degrees (Figure 4). Retentive strength of 0–15 degrees was significantly (*P* < 0.05) higher than those of 0-0, 0–5, 5-5, and 10-10 degrees, and retentive strength of 5-5 degrees was significantly (*P* < 0.05) lower than those of 0–10, 0–15, and 15-15 degrees (Table 6).

## 4. Discussion

The research hypotheses were not rejected, as the results indicated that more posterior positioning of the implants and more angulated implants exhibited higher retention in the 2IMOs. Currently there are no guidelines on selection of attachments based on their retentive abilities. This is the critical clinical aspect in 2IMO as the overdenture is usually being placed and removed multiple times in a day. The extra retentive strength may exhibit more frictional resistance onto the implant attachments daily. Greater retentive strength may also affect the longevity of the flexible matrix [6–9]. Maximum studies evaluated the retentive strengths of 2IMO after simulated function and very few evaluated the stresses at baseline without age simulation [9, 11–15]. The attachments are serviceable from the first day of their usage in mouth; hence, the present study evaluated their retentive strength without any age simulation. The retentive strengths could differ from the previous studies, up to certain extent, for the same attachment type due to the differences in the age simulation. Sia et al. [6] evaluated the effect of the different heights of pairs of the LOCATOR attachments (0, 2, 4, and 6 mm) on the retention of 2IMO after 6 months of simulated function and showed that the retention strengths of the pink LOCATOR attachment ranged from 32.3 ± 8.8 to 53.6 ± 10.2. In this study, similar pink LOCATOR attachments were used, and the similar situation of 10 mm position and 0-0 degrees indicated the strength of 53.26 ± 11.48, which was almost at the higher range of the results by Sia et al. [6]. This could be because of the difference between (with and without) age simulation methods. The immediate loading protocols are popular with the implant overdentures and it is critical to evaluate retentive strength of the attachments from the day of the implant placement and loading, especially during the osseointegration period of 4 to 6 months [18]. Hence, the evaluation of retentive strengths without insertion-removal cycles warrants the significant role in overall performance of the 2IMO.

The results of this study are in accordance with Scherer et al. [5] who indicated that the retention and stability are increased with the distal implant positions up to the second premolar. However, the range of the retentive strengths of the LOCATOR attachments was found to be at lower range. This study was in accordance with Elsyad et al. [9] who demonstrated that the retention of LOCATOR attachments was significantly affected by distal angulations of the implants and the type of nylon inserts. However, Elsyad et al. [9] used similar distal angulations of 5, 10, 15, and 20 degrees on both sides. The results of this study can be corelated with the study by Elsyad et al. [8] who evaluated the effect of labial inclinations of implants with 0, 10, 20, and 30 degrees and found out that the inclination of 30 degrees recorded the highest retention, and the inclination of 20 degrees showed the lowest retention. The lowest retention observed at 20 degrees of labial angulations was, however, not in accordance with the trend observed in this study with distal angulations. Stephens et al. [11] assessed the influence of interimplant divergence on retention of two blue Locator attachments before and after in vitro simulation of 3 to 5 years of use (5500 seating and unseating cycles). The retention of Locator pairs was not impaired by interimplant divergence of up to 20°. Retention after 5500 removal cycles was less than the initial retention in all groups. Rabbani et al. [12] evaluated the influence of mesial angulation of 10 degrees on one side (0/10) or 5 degrees on both sides (5/5). The specimens were subjected to cyclic loading to simulate 6, 12, and 18 months of clinical use (720, 1,440, or 2,160 cycles). A rapid decrease in retentive force was observed in all three models after 720 cycles for all three inserts. After 2,160 cycles, there was a significant reduction in retentive force of 59% to 70%. Kobayashi et al. [10] evaluated different attachments with implants placed at angulation of up to 12 degrees between implants and found out that the retentive forces and attachment wear were not significantly affected. This could be possibly because the interimplant angulation of 12° (6 degrees for each implant) may not be significantly greater compared with 0-0-degree angulation. This study shows that the retentive strengths of 0–5 and 5-5 degrees' angulations did not significantly differ from 0-0 degree angulation (Table 6). In general, the dissimilar angulations on both sides (0–5, 0–10, and 0–15) exhibited higher retentive strengths as compared with similar angulations (5-5, 10-10, and 15-15), respectively.

The positions of the lateral incisor, canine, and premolars may vary according to the age, sex, ethnicity, and physique of an individual. Hence, 4 different positions (5 mm, 10 mm, 15 mm, and 20 mm) were evaluated in this study. In 2IMO, parallel placement of two implants is not always possible because of anatomic variations and compromised bone volume. Hence, this study used 3 situations with similar angulations (5-5, 10-10, and 15-15 degrees) on both sides and 3 situations with dissimilar angulations by keeping left side implant at 0 degrees (0–5, 10-10, and 15-15 degrees).

This study used the method of 3D simulation which facilitated the replication of an anatomic mandible that showed better resemblance of the clinical situation than use of the casts. Previous studies have used different types of surveyors and mathematical instruments to accurately control implant positions and angulations. This study used virtual planning of the implant positions and angulations which had better accuracy than physically controlled placements. In this study, different hole diameters and lengths were first studied to select the most appropriate hole dimension for placement of the implants sized 4.3 × 13 mm in the 3D-printed models. This was to place the implants firmly and accurately and to minimize the errors related to virtual model dimensions, printing resin material shrinkage, and different printing stages. Previous studies have not simulated the mucosal layer onto the study models [5–10]. While dislodging the 2IMO under universal testing machine, there is a possibility that the denture may get tilted to any side before completely being separated from both attachments and hard cast surface may influence the retentive strength measurements up to a certain extent. Hence, this tilting may result in the compression of the denture flange on opposite side. This compression on hard cast surface may provide additional leverage effect and may affect the final retentive strength. Hence, soft-tissue simulation in such in vitro studies is important to minimize the measurement errors. This study has simulated the 2 mm uniform mucosal layer and could be another advancing methodology feature.

When providing a 2-implant mandibular overdenture, more posteriorly positioned implants as well as more angulated implants exhibit higher retention of the overdentures. Clinician must be aware of these variations in retentive strengths related to different implant positions and angulations so that the appropriate strength of the flexible matrix can be selected to optimize the overall retention of the overdenture.

Biomaterials used to fabricate the retentive elements also play vital role in influencing the retention of the overdenture. Chindarungruangrat et al. [13] studied the retentive force of retentive element materials using three retentive element materials: nylon, polyetheretherketone (PEEK), and polyvinyl siloxane (PVS). The retentive force (N) was measured before thermocycling and at 2500, 5000, and 10,000 cycles after thermocycling and it was concluded that the retentive element materials tend to lose their retentive capability because of thermal undulation and water dispersion. Nylon and PEEK showed a higher rate of retention loss than polyvinyl siloxane. Yılmaz et al. [14] compared the retention forces of implant overdenture patrices (ball, bar, and TiSi.snap) to conventional (O-ring, metal housing, and clip) and polyvinyl siloxane- (PVS-) based silicone matrix materials. Loss of retention occurred in all the attachment systems at the end of 3,650 cycles. Further studies are advocated using different retentive elements like PEEK and polyvinyl siloxane. Wakam et al. [15] carried out systematic review of 14 clinical and 31 in vitro studies regarding the retention, wear, and maintenance of attachments used clinically or in vitro specifically for 1IMO or 2IMO. They have concluded that the plastic retention devices wear out faster and more significantly than metal ones. Since there are varieties of materials used to fabricate the retentive elements, further clinical and in vitro studies are advocated evaluating their effectiveness.

This study used only different combinations of distal angulations (as a frequent clinical possibility) under vertical dislodging force. These can be considered as limitations of the study. Future in vitro studies with different 2IMO simulations with different angulations under anterioposterior or oblique dislodging force can be planned. Future clinical studies are also recommended on 2IMO evaluating patient-reported outcomes and clinical parameters using different unsplinted attachments.

## 5. Conclusions

Based on the findings of this 3D-printed-simulation study, the following conclusions were drawn. Retentive strength of the 2IMO increased by increasing the distance of the implants from midline. The retentive strength progressively increased from 5 to 10 to 15 to 20 mm from midline represented as lateral incisor, canine, and premolar positions. Retentive strength of the 2IMO increased with increase in distal angulations of the implants when placed at 10 mm from midline represented as a canine position.

## Figures and Tables

**Table 1:** Descriptive statistics of different implant positions.

Implant position	*N*	Mean ± std. deviation	Std. error	95% confidence interval		Minimum	Maximum
				Lower bound	Upper bound		
5 mm	12	49.64 ± 8.27	2.38708	44.3894	54.8973	38.29	65.37
10 mm	12	53.26 ± 11.48	3.31278	45.9670	60.5497	31.69	69.07
15 mm	12	60.24 ± 12.31	3.55448	52.4141	68.0609	44.68	85.67
20 mm	12	64.80 ± 6.78	1.95648	60.4988	69.1112	54.60	77.04
Total	48	56.99 ± 11.34	1.63733	53.6922	60.2799	31.69	8. 567

**Table 2:** ANOVA with different implant positions.

	Sum of squares	d*f*	Mean square	*F*	*P* value
Between groups	1674.231	3	558.077	5.614	0.002
Within groups	4373.795	44	99.404		
Total	6048.027	47			

**Table 3:** Pairwise comparison of retentive strengths with different implant positions.

Positions (mm) (*I*)	Positions (mm) (*J*)	Mean difference (*I* − *J*)	*P* value	95% confidence interval	
				Lower bound	Upper bound
5	10	−3.61500	0.811	−14.4827	7.2527
	15	−10.59417	0.058	−21.4619	0.2736
	20	−15.16167^*∗*^	0.003	−26.0294	−4.2939
10	5	3.61500	0.811	−7.2527	14.4827
	15	−6.97917	0.328	−17.8469	3.8886
	20	−11.54667^*∗*^	0.033	−22.4144	−0.6789
15	5	10.59417	0.058	−0.2736	21.4619
	10	6.97917	0.328	−3.8886	17.8469
	20	−4.56750	0.678	−15.4352	6.3002
20	5	15.16167^*∗*^	0.003	4.2939	26.0294
	10	11.54667^*∗*^	0.033	0.6789	22.4144
	15	4.56750	0.678	−6.3002	15.4352

^*∗*^The mean difference is significant at the 0.05 level.

**Table 4:** Descriptive statistics of different angulations.

Angulations	*N*	Mean ± std. deviation	Std. error	95% confidence interval		Minimum	Maximum
				Lower bound	Upper bound		
0-0	12	53.26 ± 11.48	3.31278	45.9670	60.5497	31.69	69.07
0–5	12	54.96 ± 8.25	2.38238	49.7139	60.2011	40.20	67.13
5-5	12	48.20 ± 15.59	4.50172	38.2943	58.1107	31.00	72.79
0–10	12	66.18 ± 14.09	4.06707	57.2234	75.1266	43.58	83.06
10-10	12	57.71 ± 7.62	2.19976	52.8717	62.5550	44.67	74.63
0–15	12	77.38 ± 10.33	2.98146	70.8204	83.9446	56.37	92.47
15-15	12	66.00 ± 17.54	5.06251	54.8608	77.1459	35.61	88.42
Total	84	60.53 ± 15.26	1.66497	57.2159	63.8391	31.00	92.47

**Table 5:** ANOVA with different implant angulations.

	Sum of squares	d*f*	Mean square	*F*	Sig.
Between groups	7075.931	6	1179.322	7.412	0.000
Within groups	12251.428	77	159.109		
Total	19327.358	83			

**Table 6:** Pairwise comparison of retentive strengths with different implant angulations.

Angle (*I*)	Angle (*J*)	Mean difference (*I* − *J*)	*P* value	95% confidence interval	
				Lower bound	Upper bound
0-0	0–5	−1.69917	1.000	−17.2904	13.8920
	5-5	5.05583	0.956	−10.5354	20.6470
	0–10	−12.91667	0.171	−28.5079	2.6745
	10-10	−4.45500	0.977	−20.0462	11.1362
	0–15	−24.12417^*∗*^	0.000	−39.7154	−8.5330
	15-15	−12.74500	0.183	−28.3362	2.8462
0–5	0-0	1.69917	1.000	−13.8920	17.2904
	5-5	6.75500	0.844	−8.8362	22.3462
	0–10	−11.21750	0.319	−26.8087	4.3737
	10-10	−2.75583	0.998	−18.3470	12.8354
	0–15	−22.42500^*∗*^	0.001	−38.0162	−6.8338
	15-15	−11.04583	0.338	−26.6370	4.5454
5-5	0-0	−5.05583	0.956	−20.6470	10.5354
	0–5	−6.75500	0.844	−22.3462	8.8362
	0–10	−17.97250^*∗*^	0.014	−33.5637	−2.3813
	10-10	−9.51083	0.521	−25.1020	6.0804
	0–15	−29.18000^*∗*^	0.000	−44.7712	−13.5888
	15-15	−17.80083^*∗*^	0.015	−33.3920	−2.2096
0–10	0-0	12.91667	0.171	−2.6745	28.5079
	0–5	11.21750	0.319	−4.3737	26.8087
	5-5	17.97250^*∗*^	0.014	2.3813	33.5637
	10-10	8.46167	0.655	−7.1295	24.0529
	0–15	−11.20750	0.320	−26.7987	4.3837
	15-15	0.17167	1.000	−15.4195	15.7629
10-10	0-0	4.45500	0.977	−11.1362	20.0462
	0–5	2.75583	0.998	−12.8354	18.3470
	5-5	9.51083	0.521	−6.0804	25.1020
	0–10	−8.46167	0.655	−24.0529	7.1295
	0–15	−19.66917^*∗*^	0.005	−35.2604	−4.0780
	15-15	−8.29000	0.676	−23.8812	7.3012
0–15	0-0	24.12417^*∗*^	0.000	8.5330	39.7154
	0–5	22.42500^*∗*^	0.001	6.8338	38.0162
	5-5	29.18000^*∗*^	0.000	13.5888	44.7712
	0–10	11.20750	0.320	−4.3837	26.7987
	10-10	19.66917^*∗*^	0.005	4.0780	35.2604
	15-15	11.37917	0.303	−4.2120	26.9704
15-15	0-0	12.74500	0.183	−2.8462	28.3362
	0–5	11.04583	0.338	−4.5454	26.6370
	5-5	17.80083^*∗*^	0.015	2.2096	33.3920
	0–10	−0.17167	1.000	−15.7629	15.4195
	10-10	8.29000	0.676	−7.3012	23.8812
	0–15	−11.37917	0.303	−26.9704	4.2120

^*∗*^The mean difference is significant at the 0.05 level.

## Data Availability

The data used to support the findings of this study are available from the corresponding author upon request.
